# Effects of Different Supplemental Levels of *Eucommia ulmoides* Leaf Extract in the Diet on Carcass Traits and Lipid Metabolism in Growing–Finishing Pigs

**DOI:** 10.3389/fvets.2021.828165

**Published:** 2022-02-07

**Authors:** Yuhuan Yang, Fengna Li, Qiuping Guo, Wenlong Wang, Lingyu Zhang, Yunju Yin, Saiming Gong, Mengmeng Han, Yulong Yin

**Affiliations:** ^1^College of Animal Science and Technology, Hunan Agricultural University, Changsha, China; ^2^Hunan Provincial Key Laboratory of Animal Nutritional Physiology and Metabolic Process, National Engineering Laboratory for Pollution Control and Waste Utilization in Livestock and Poultry Production, Key Laboratory of Agro-Ecological Processes in Subtropical Region, Hunan Provincial Engineering Research Center for Healthy Livestock and Poultry Production, Scientific Observing and Experimental Station of Animal Nutrition and Feed Science in South-Central, Ministry of Agriculture, Institute of Subtropical Agriculture, Chinese Academy of Sciences, Changsha, China; ^3^College of Advanced Agricultural Sciences, University of Chinese Academy of Sciences, Beijing, China

**Keywords:** *Eucommia ulmoides* leaf extract, DLY growing-finishing pigs, growth performance, carcass trait, lipid metabolism, AMPK-ACC signal pathway

## Abstract

This study examined the effects of dietary *Eucommia ulmoides* leaf extract (ELE) supplements on carcass traits and lipid metabolism in growing–finishing pigs. A total of 144 crossbred (Duroc × Landrace × Yorkshire) piglets with an average initial weight of 10.11 ± 0.03 kg were randomly allotted to four treatment groups, each with six replicates and six piglets per replicate. Each group of pigs was fed a basal diet or a diet supplemented with increasing levels of ELE (0.1, 0.2, or 0.3%). The results showed that adding ELE had no negative effect on the growth performance of pigs. Dietary supplements of 0.1% ELE significantly increased carcass weight (*p* < 0.01), dressing percentage (*p* < 0.01), carcass length (*p* < 0.05), and eye muscle area (*p* < 0.05). Compared with the control group, a 0.2% ELE supplement significantly increased (*p* < 0.01) the levels of adiponectin, insulin-like growth factor 1, and hormone-sensitive lipase and lipoprotein lipase activity in the serum. Histological examination showed that ELE inhibited fat deposition in the backfat tissue. Lipid metabolism-related biochemical indices and mRNA expression levels were improved after supplementing diets with ELE. Moreover, all three levels of ELE dramatically upregulated (*p* < 0.05) the protein levels of p-AMPK-α and p-ACC. In summary, adding ELE to pig diets could improve the carcass traits of growing–finishing pigs and exert a lipid-lowering effect by activating the AMPK-ACC pathway and regulating mRNA expression levels related to lipid metabolism. Supplementing the diet with 0.1–0.2% ELE is the optimal range to reduce fat deposition in pig backfat tissue.

## Introduction

Obesity is becoming one of the most important health problems in several countries, affecting scores of people, as it increases the risk of various diseases, such as fatty liver, diabetes, and coronary heart disease (1). In recent years, an antiobesity effect of *Eucommia ulmoides* has been supported by an increasing number of studies. Two prevenient studies have reported that *Eucommia ulmoides* improved hyperglycemia in diabetic rats (2) and type 2 diabetes patients (3). Moreover, *Eucommia ulmoides* promoted the recovery of lipid metabolism disorders caused by a high-fat diet in rats (4).

*Eucommia ulmoides* (Chinese: Duzhong), also known as Gutta-percha tree, Sixian, and Sizhong, is a perennial deciduous tree of the Eucommiaceae family (5). *Eucommia ulmoides* is widely distributed in China, with a high annual yield (6). Its medicinal history can be traced back thousands of years and is now widely used in clinics (5). *Eucommia ulmoides* is rich in lignans, iridoid terpenoids, flavonoids, polysaccharides, and other active components, with antihypertensive, hypoglycemic, anti-inflammatory, liver protection, antitumor, and other pharmacological effects (5, 7, 8). Studies into the potential of *Eucommia ulmoides* as a feed supplement in Chinese herbal medicine have been gradually developed. Previous studies focused on the effects of *Eucommia ulmoides* leaf and its extracts on growth performance and antioxidant activity in pigs (9–11). However, at present, there are few studies on the effect of *Eucommia ulmoides* leaf extracts (ELE) on lipid metabolism in growing–finishing pigs, and the optimal supplement level is also unknown. Since there are many similarities between pigs and humans in terms of structure and function, the effect of ELE as a dietary supplement in pigs can be used as a model for the study of human nutrition and metabolism (12).

Following from previous research, we added dietary supplements of 0, 0.1, 0.2, or 0.3% ELE to growing–finishing pig diets and recorded the effects on growth performance, carcass traits, and lipid metabolism. This provides a basis for the wider application of ELE in animal husbandry and reducing the incidence of human obesity.

## Materials and Methods

### Preparation of ELE

ELE were purchased from Zhangjiajie Hengxing Biotechnology Co., Ltd. (Zhangjiajie, China). Data provided by the company show that the main active ingredients include 5% chlorogenes, 8% EL flavonoids, and 20% EL polysaccharides.

### Animals and Diets

The animal experiments were approved by the Committee on Animal Care of the Institute of Subtropical Agriculture, Chinese Academy of Sciences. A total of 144 crossbred barrows (Duroc × Landrace × Yorkshire, DLY, 10.11 ± 0.03 kg) were randomly divided into four treatments, six replicates in each treatment, and six pigs in each replicate. The experimental diets were as follows: (1) control diet; (2) control diet + 0.1% ELE; (3) control diet + 0.2% ELE; (4) control diet + 0.3% ELE. All the growing–finishing pigs were raised in pens and had *ad libitum* access to diets and clean drinking water. All pigs were weighed when the pigs in the control group weigh 10, 30, 70, and 115 kg, and feed intake was recorded every week to calculate the average daily gain (ADG), average daily feed intake (ADFI), and the ratio of feed to gain (F/G). The experiment used a corn–soybean meal diet referred to NRC (1998, 2012). The ingredients and nutritional composition of basal diet are shown in Table 1.

**Table 1:** Ingredients and nutritional composition of basic diets.

**Ingredients (%)**	**Dietary treatment**		
	**10–30 kg**	**30–70 kg**	**70–115 kg**
Corn	63.70	58.60	67.00
Soybean meal	19.80	29.00	23.76
Dried whey	4.30	–	–
Wheat bran	–	7.80	6.00
Fish meal	9.00	–	–
Soybean oil	0.80	1.55	0.88
Lys	0.38	0.18	0.01
Met	0.10	0.00	0.00
Thr	0.09	0.01	0.00
Trp	0.01	0.00	0.00
CaHPO_4_	0.00	0.69	0.50
Limestone	0.52	0.87	0.55
Salt	0.30	0.30	0.30
Premix^a^	1.00	1.00	1.00
Total	100.00	100.00	100.00
**Nutrient content (%)**
DE^b^ (MJ/kg)	14.60	14.20	14.20
CP	20.27	18.27	16.30
Total Lys	1.52	1.15	0.88
Total (Met + Cys)	0.79	0.61	0.55
Total Thr	0.94	0.77	0.68
Total Trp	0.26	0.25	0.21
Total Ca	0.69	0.60	0.52
Total P	0.57	0.51	0.45

a *Supplied per kg of diet (10–30 kg): vitamin A, 18,000 IU; vitamin D_3_, 5,000 IU; vitamin E, 40 IU; vitamin K_3_, 4 mg; vitamin B_1_, 6 mg; vitamin B_2_, 12 mg; vitamin B_6_, 6 mg; vitamin B_12_, 0.05 mg; biotin, 0.2 mg; folic acid, 2 mg; niacin, 50 mg; D-calcium pantothenate, 25 mg; Cu (as copper sulfate), 20 mg; Fe (as ferrous sulfate), 90 mg; Mn (as manganese oxide), 15 mg; Zn (as zinc oxide), 80 mg; I (as potassium iodide), 0.3 mg; and Se (as sodium selenite), 0.3 mg. Supplied per kg of diet (30–115 kg): vitamin A, 15,000 IU; vitamin D_3_, 3,000 IU; vitamin E, 40 IU; vitamin K_3_, 4 mg; vitamin B_1_, 3 mg; vitamin B_2_, 10 mg; vitamin B_6_, 4 mg; vitamin B_12_, 0.03 mg; biotin, 0.2 mg; folic acid, 2 mg; niacin, 35 mg; D-calcium pantothenate, 20 mg; Cu (as copper sulfate), 15 mg; Fe (as ferrous sulfate), 80 mg; Mn (as manganese oxide), 15 mg; Zn (as zinc oxide), 70 mg; I (as potassium iodide), 0.5 mg; and Se (as sodium selenite), 0.3 mg*.

b *Calculated value for DE*.

### Sample Collection

At the end of the trial, all the pigs were fasted overnight (12 h), and one or two pigs of each replicate with average final body weight was selected (8 pigs/treatment) to slaughter by electrical stunning in a commercial abattoir. Before slaughter, blood samples were collected into a plain tube and placed at room temperature for 30 min, then centrifuged at 3,000 × g for 10 min at 4°C. Serum was collected and stored at −80°C for further analysis (13). The backfat samples were immediately excised and stored at −20°C for determination of the chemical composition or placed in liquid N_2_ and then stored at −80°C for the analysis of quantitative real-time PCR. Fresh samples of backfat (1 cm^3^) were fixed in paraformaldehyde fixative for paraffin sections and hematoxylin and eosin staining.

### Carcass Trait Analysis

At slaughter, the carcass and the left side of carcass were weighted so that slaughter rate could be calculated. Other carcass traits including carcass length (carcass straight length and carcass slant length), average backfat thickness (the 3rd−4th lumbar spine, the 10th−11th lumbar spine, and the last rib), and loin–eye area were measured from the left side of the carcass. The left side of the carcass was split up into skeletal muscle and fat as previously described (14). The fat mass rate percentage and lean mass percentage were weighed and calculated.

### Serum Biochemical Index Measurements

Total protein (TP), albumin (ALB), urea nitrogen (BUN), blood glucose (GLU), total cholesterol (TC), triglyceride (TG), high-density lipoprotein cholesterol (HDL-C), low-density lipoprotein cholesterol (LDL-C), and very low-density lipoprotein cholesterol (VLDL-C) in serum were measured with cobas C311 Analyzer (Roche Diagnostics, Basel, Switzerland) and commercial kits (Leadman Biotech Limited, Beijing, China) as specified by the manufacturer.

### Measurement of Serum Cytokine Levels

The concentrations of leptin (LEP), adiponectin (ADPN), insulin (INS), and insulin-like growth factor 1 (IGF-1) were performed by using ELISA kits (Changsha Aoji Biotechnology Co., Ltd., Changsha, China).

### Measurement of Serum Enzyme Activity

The activity of acetyl-coa carboxylase (ACC), hormone sensitive lipase (HSL), lipoprotein lipase (LPL), adipose triacylglyceride lipase (ATGL), and acyl CoA cholesterol acyltransferase (ACAT) in serum was detected by ELISA kits (Changsha Aoji Biotechnology Co., Ltd., Changsha, China).

### Backfat Tissue Histological Analysis

The mean cross-sectional area and quantity of adipocyte in backfat tissue were measured by classic hematoxylin and eosin staining. Serial tissue sections of 4 μm were sliced using a paraffin slicer (RM 2016, Shanghai Leica Instrument Co., Ltd., Shanghai, China). The slices were dyed with hematoxylin dye solution for 3–5 min, washed with tap water and dehydrated with 85 and 95% gradient alcohol for 5 min, respectively, then dyed with eosin dye solution for 5 min, dehydrated with absolute ethanol, and finally sealed with neutral gum. The stained slides are scanned with a panoramic slice scanner of Pannoramic DESK/MIDI/250/1000 (3DHISTECH, Budapest, Hungary), the scanned slices are opened with CaseViewer 2.4 software (3DHISTECH, Hungary), the field of view is intercepted, and Image-Pro Plus 6.0 (Media Cybernetics, Rockville, MD, USA) is used for calculation and analysis.

### Total RNA Isolation and Quantitative Real-Time PCR Analysis

Total RNA isolation and real-time quantitative PCR were conducted as previously described (15). In brief, total RNA was extracted from backfat tissue samples using TRIzol Reagent (Hunan Aikerui Bioengineering Co., Ltd., Changsha, China). The purity of the total RNA was verified using a NanoDrop ND2000 (NanoDrop Technologies Inc., Wilmington, DE, USA) at 260 and 280 nm, and the OD260/OD280 ratios of the RNA samples were all between 1.8 and 2.0. The total RNA was treated with DNase I (Hunan Aikerui Bioengineering Co., Ltd., Changsha, China) to remove DNA and reverse transcribed to complementary deoxyribonucleic acid (cDNA) using Evo M-MLV RT Kits with gDNA clean for qPCR (Hunan Aikerui Bioengineering Co., Ltd., Changsha, China) following the manufacturer's protocol. Quantitative real-time PCR was performed using an ABI 7900HT Real-Time PCR system (Applied Biosystems, Branchburg, NJ, USA) with SYBR Green Premix Pro Taq HS qPCR Kits (Hunan Aikerui Bioengineering Co., Ltd., Changsha, China). The PCR system consisted of 5 μl SYBR Green Pro Taq HS Premix, 2 μl cDNA, 2.2 μl RNase-free water, and 0.4 μl primer pairs (forward and reverse) in a total volume of 10 μl. The PCR protocols included one cycle at 95°C for 30 s, 40 cycles at 95°C for 5 s, and 60°C for 30 s. Glyceraldehyde-3-phosphate dehydrogenase (GAPDH) was used as the endogenous control gene to normalize the expression of target genes according to the comparative Ct method as follows: 2 ^−^ΔΔCt (ΔΔ*Ct* = ΔCt _geneofinterest_−Δ*Ct*_GAPDH_) (16). Primer sequences are shown in the Table 2.

**Table 2:** Primers used for quantitative real-time PCR.

**Genes^a^**	**Primers**	**Sequences (5^′^to 3^′^)**	**Product size, bp**
ACC	Forward	AGCAAGGTCGAGACCGAAAG	169
	Reverse	TAAGACCACCGGCGGATAGA	
FAS	Forward	CTACCTTGTGGATCACTGCATAG	114
	Reverse	GGCGTCTCCTCCAAGTTCTG	
SREBP-1c	Forward	GCGACGGTGCCTCTGGTAGT	218
	Reverse	CGCAAGACGGCGGATTTA	
HSL	Forward	CACAAGGGCTGCTTCTACGG	167
	Reverse	AAGCGGCCACTGGTGAAGAG	
LPL	Forward	CTCGTGCTCAGATGCCCTAC	148
	Reverse	GGCAGGGTGAAAGGGATGTT	
ATGL	Forward	TCACCAACACCAGCATCCA	95
	Reverse	GCACATCTCTCGAAGCACCA	
CPT1B	Forward	GACAAGTCCTTCACCCTCATCGC	170
	Reverse	GGGTTTGGTTTGCCCAGACAG	
PPAR γ	Forward	CCAGCATTTCCACTCCACACTA	124
	Reverse	GACACAGGCTCCACTTTGATG	
AMPK α	Forward	GCATAGTTGGGTGAGCCACA	105
	Reverse	CCTGCTTGATGCACACATGA	
FATP1	Forward	ACCACTCCTACCGCATGCAG	78
	Reverse	CCACGATGTTCCCTGCCGAGT	
FAT/CD36	Forward	CTGGTGCTGTCATTGGAGCAG	160
	Reverse	CTGTCTGTAAACTTCCGTGCCTGTT	
FABP4	Forward	CAGGAAAGTCAAGAGCACCA	227
	Reverse	TCGGGACAATACATCCAACA	
GAPDH	Forward	CAAAGTGGACATTGTCGCCATCA	123
	Reverse	AGCTTCCCATTCTCAGCCTTGACT	

a *ACC, acetyl CoA carboxylase; FAS, fatty acid synthase; HSL, hormone-sensitive lipase; LPL, lipoprotein lipase; ATGL, adipose triacylglyceride lipase; CPT1B, carnitine palmitoyl transferase 1B; FATP1, fatty acid transport protein 1; FAT/CD36, fatty acid translocase; FABP4, fatty acid-binding protein 4; SREBP1c, sterol regulatory element-binding protein-1c; PPAR γ, translocase peroxisome proliferator-activated receptor γ; AMPK α, adenosine monophosphate-activated protein kinase α*.

### Western Blot Analysis

An appropriate amount of backfat tissue sample was weighed and added to RIPA lysate for ice lysis, and then BCA protein assay kits (Beyotime Biotechnology, Shanghai, China) were used to measure the protein concentration. Next, SDS-PAGE electrophoresis was carried out. Firstly, the glass plate was cleaned, and then the glue with an appropriate concentration was prepared according to the protein concentration of the sample. The loading amount was calculated, and β-mercaptoethanol was added to the equal-volume buffer and 1/10-volume buffer, mixed well, put into the Mastercycler nexus PCR instrument (Eppendorf, Hamburg, Germany), and mixed well. After adding samples, electrophoresis was carried out, and then the membrane was transferred. After sealing the membrane, the primary antibody and secondary antibody were incubated for color development.

### Statistical Analysis

All experimental data were analyzed using one-way analysis of variance (ANOVA) of SPSS (version 26.0, SPSS Inc., Chicago, IL, USA), and then the Duncan multiple-comparison test was performed. Results were expressed as mean and SEM, *p* < 0.05 was considered significant, and 0.05 ≤ *p* < 0.10 was considered as trend.

## Results

### Growth Performance

Table 3 shows that from 10 to 30 kg, there was no significant difference (*p* > 0.05) in ADG, ADFI, or F/G with increasing levels of ELE supplements. At the 10–70-kg stage, ADG was higher in the group supplemented with 0.1% ELE (*p* < 0.05) than in the other groups. F/G was lower in the 0.1% ELE group (*p* < 0.05) than in the other groups, but there was no dramatic discrepancy (*p* > 0.05) compared with the control group. Adding 0.2 or 0.3% ELE to the diet could markedly improve ADFI (*p* < 0.05). Over the whole period of the experiment, ADFI was higher (*p* < 0.05) in the 0.1% ELE group than in the 0.3% group, but there were no observable change in ADG or F/G among the different treatments.

**Table 3:** Growth performance of growing and growing–finishing pigs fed the diets with various levels of ELE.

**Item^1^**	**ELE^2^ levels, %**				**SEM**	***p-value***
	**0**	**0.1**	**0.2**	**0.3**		
10–30 kg
Initial weight, kg	10.08	10.11	10.12	10.11	0.03	0.76
Final weight, kg	29.23	28.76	29.18	28.77	0.27	0.46
ADG, kg·day^−1^	0.54	0.54	0.55	0.54	0.01	0.83
ADFI, kg·day^−1^	0.90	0.93	0.93	0.91	0.01	0.07
F/G	1.68	1.72	1.71	1.71	0.02	0.37
10–70 kg
Final weight, kg	69.65	71.60	68.24	68.83	1.13	0.20
ADG, kg·day^−1^	0.63^ab^	0.65^a^	0.62^b^	0.61^b^	0.01	0.04
ADFI, kg·day^−1^	1.41^b^	1.45^a^	1.46^a^	1.42^b^	0.01	0.02
F/G	2.26^ab^	2.22^b^	2.32^a^	2.32^a^	0.02	0.02
10–115 kg
Final weight, kg	114.33	116.18	115.33	114.95	1.51	0.39
ADG, kg·day^−1^	0.69	0.71	0.70	0.68	0.01	0.44
ADFI, kg·day^−1^	1.96^ab^	1.99^a^	1.96^ab^	1.95^b^	0.01	0.06
F/G	2.85	2.82	2.82	2.88	0.03	0.39

ab *Different superscript letters on the same line are significant differences (p < 0.05) (n = 6)*.

1 *ADG, average daily weight gain; ADFI, average daily feed intake; F/G, the ratio of feed intake to body weight gain*.

2 *ELE, Eucommia ulmoides leaf extract*.

### Carcass Trait

Table 4 shows that carcass weight (*p* < 0.05), slaughter rate (*p* < 0.01), and carcass straight length (*p* < 0.05) in the 0.1% ELE group were markedly higher than those in the other three groups. Meanwhile, compared with the control group, dietary supplemented with 0.1% ELE observably aggrandized the loin–eye area (*p* < 0.05) of growing–finishing pigs. In addition, adding 0.3% ELE notably increased the lean meat rate (*p* < 0.01) of growing–finishing pigs compared with the other three groups.

**Table 4:** Carcass trait of growing–finishing pigs fed the diets with various levels of ELE.

**Item**	**ELE^1^ levels, %**				**SEM**	***p-value***
	**0**	**0.1**	**0.2**	**0.3**		
Carcass weight, kg	82.63^b^	87.00^a^	84.27^b^	83.73^b^	0.93	0.02
Slaughter rate, %	73.37^b^	75.39^a^	73.46^b^	72.85^b^	0.39	<0.01
Carcass straight length, cm	95.91^b^	98.64^a^	95.64^b^	95.74^b^	0.79	0.03
Carcass slant length, cm	82.29	83.45	82.14	82.64	0.62	0.50
Average backfat thickness, mm	25.63	24.94	24.46	23.74	1.11	0.55
Loin–eye area, cm^2^	27.17^b^	31.60^a^	29.70^ab^	30.96^a^	0.94	0.01
Lean mass percentage, %	55.00^b^	55.54^b^	55.46^b^	58.40^a^	0.75	<0.01
Fat mass percentage, %	16.19	15.37	15.65	16.00	0.56	0.85

ab *Different superscript letters on the same line are significant differences (p < 0.05) (n = 8)*.

1 *ELE, Eucommia ulmoides leaf extract*.

### Serum Biochemical Indexes

Table 5 shows that adding ELE in the diet memorably descended the content of TP (*p* < 0.05), TG (*p* < 0.05), and VLDL-C levels (*p* < 0.01) compared with the control group; meanwhile, it increased serum ALB (*p* < 0.01) and HDL-C levels (*p* < 0.01).

**Table 5:** Effects of dietary ELE on serum biochemical indexes of growing–finishing pigs.

**Item^1^**	**ELE^2^** **levels, %**				**SEM**	***p-value***
	**0**	**0.1**	**0.2**	**0.3**		
TP, g·L^−1^	74.39^a^	72.90^ab^	72.54^ab^	70.61^b^	0.84	0.03
ALB, g·L^−1^	52.67^a^	55.39^a^	53.41^a^	46.64^b^	1.05	<0.01
BUN, mmol·L^−1^	5.20^ab^	5.44^ab^	6.03^a^	4.63^b^	0.28	0.01
GLU, mmol·L^−1^	6.36	6.45	6.30	6.27	0.21	0.93
TG, mmol·L^−1^	0.58^a^	0.46^b^	0.49^ab^	0.43^b^	0.04	0.03
TC, mmol·L^−1^	2.73	3.05	2.78	2.99	0.14	0.30
LDL-C, mmol·L^−1^	1.86	1.92	1.79	2.05	0.09	0.25
VLDL-C, mmol·L^−1^	15.11^a^	14.11^ab^	11.81^c^	12.60^bc^	0.57	<0.01
HDL-C, mmol·L^−1^	0.56^b^	0.84^a^	0.71^a^	0.55^b^	0.05	<0.01

a−c *Different superscript letters on the same line are significant differences (p < 0.05) (n = 8)*.

1 *TP, total protein; ALB, albumin; BUN, urea nitrogen; Glu, blood glucose; TC, total cholesterol; TG, triglyceride; HDL-C, high-density lipoprotein cholesterol; LDL-C, low-density lipoprotein cholesterol; VLDL-C, very low-density lipoprotein cholesterol*.

2 *ELE, Eucommia ulmoides leaf extract*.

### Serum Cytokine Levels

Table 6 shows that dietary supplementation with 0.2% ELE could signally elevate the levels of ADPN (*p* < 0.01) and IGF-1 (*p* < 0.01) in serum. Serum leptin and insulin levels were not changed dramatically (*p* > 0.05) among the groups.

**Table 6:** Effects of dietary ELE on serum cytokine levels of growing–finishing pigs.

**Item^1^**	**ELE^2^ levels, %**				**SEM**	***p-value***
	**0**	**0.1**	**0.2**	**0.3**		
LEP, ng·mL^−1^	11.60	10.68	10.06	11.90	0.62	0.16
ADPN, μg·mL^−1^	25.45^b^	26.75^b^	35.35^a^	24.54^b^	1.62	<0.01
INS, mIU·L^−1^	26.80	26.08	24.40	25.82	1.91	0.85
IGF-1, ng·mL^−1^	371.34^b^	449.79^b^	604.92^a^	543.78^a^	31.40	<0.01

ab *Different superscript letters on the same line are significant differences (p < 0.05) (n = 8)*.

1 *LEP, leptin; ADPN, adiponectin; INS, insulin; IGF-1, insulin-like growth factor 1*.

2 *ELE, Eucommia ulmoides leaf extract*.

### Activity of Enzymes Related to Lipid Metabolism

The activities of HSL, LPL, and ACAT first increased and then decreased as the level of ELE increased in the diet (Table 7). ACC activity was lower (*p* < 0.01) at 0.1% ELE than at other levels. Compared with the control group, diet supplemented with 0.1% and 0.2% ELE notably enhanced (*p* < 0.01) HSL and LPL activities. Besides, the ATGL activity of the 0.1% ELE group exceeded (*p* < 0.05) that of the other two treatment groups, but there was no marked difference compared with the control group. In addition, ACAT activity was no marked discrepancy among the four groups.

**Table 7:** Effects of different levels of ELE on key enzyme activity-related lipid metabolism in backfat tissue of growing–finishing pigs.

**Item^1^**	**ELE^2^ levels, %**				**SEM**	***p-value***
	**0**	**0.1**	**0.2**	**0.3**		
ACC, U·L^−1^	27.57^b^	22.21^c^	26.40^b^	33.16^a^	1.27	<0.01
HSL, U·L^−1^	843.40^b^	1189.28^a^	1287.42^a^	975.86^b^	57.32	<0.01
LPL, U·L^−1^	454.19^c^	603.63^ab^	682.70^a^	556.42^b^	28.38	<0.01
ATGL, mIU·mL^−1^	315.63^ab^	345.28^a^	290.35a^b^	263.12^b^	23.06	0.01
ACAT, U·L^−1^	85.94	86.71	94.64	87.97	5.03	0.60

a−c *Different superscript letters on the same line are significant differences (p < 0.05) (n = 8)*.

1 *ACC, acetyl CoA carboxylase; HSL, hormone-sensitive lipase; LPL, lipoprotein lipase; ATGL, adipose triacylglyceride lipase; ACAT, acyl CoA cholesterol acyltransferase*.

2 *ELE, Eucommia ulmoides leaf extract*.

### Mean Cross-Sectional Area and Quantity of Adipocyte in Backfat Tissue

**Figure 1:**
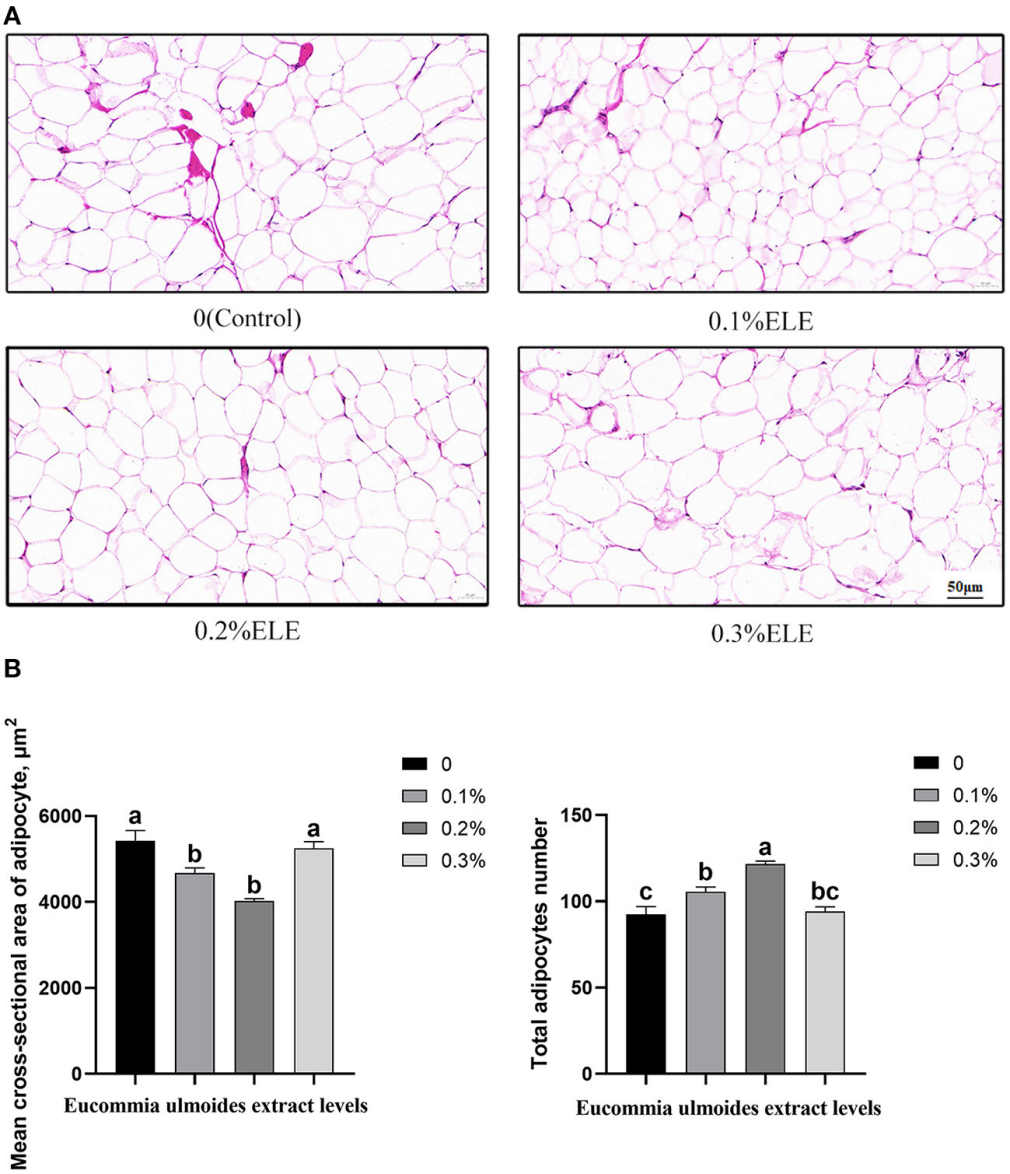
Histological analysis of the mean cross-sectional area and quantity of adipocyte of growing–finishing pigs fed the diets of different levels of *Eucommia ulmoides* leaf extract (ELE). **(A)** Representative cross-sectional HE staining photos of adipocytes in backfat tissue (magnification ×100, bar = 50 μm). **(B)** Quantitative analysis of adipocyte number in backfat tissue. Data are expressed as means ± SEM. ^a−c^Values with different letters are significantly different among dietary ELE treatments (*p* < 0.05) (*n* = 4).

Figure 1 shows that all the supplementary levels of ELE significantly decreased (*p* < 0.05) the mean cross-sectional area of adipocytes and increased (*p* < 0.05) the total number of adipocytes in backfat tissue.

### Relative mRNA Expression Levels of the Key Genes Related to Lipid Metabolism in Backfat Tissue

**Figure 2:**
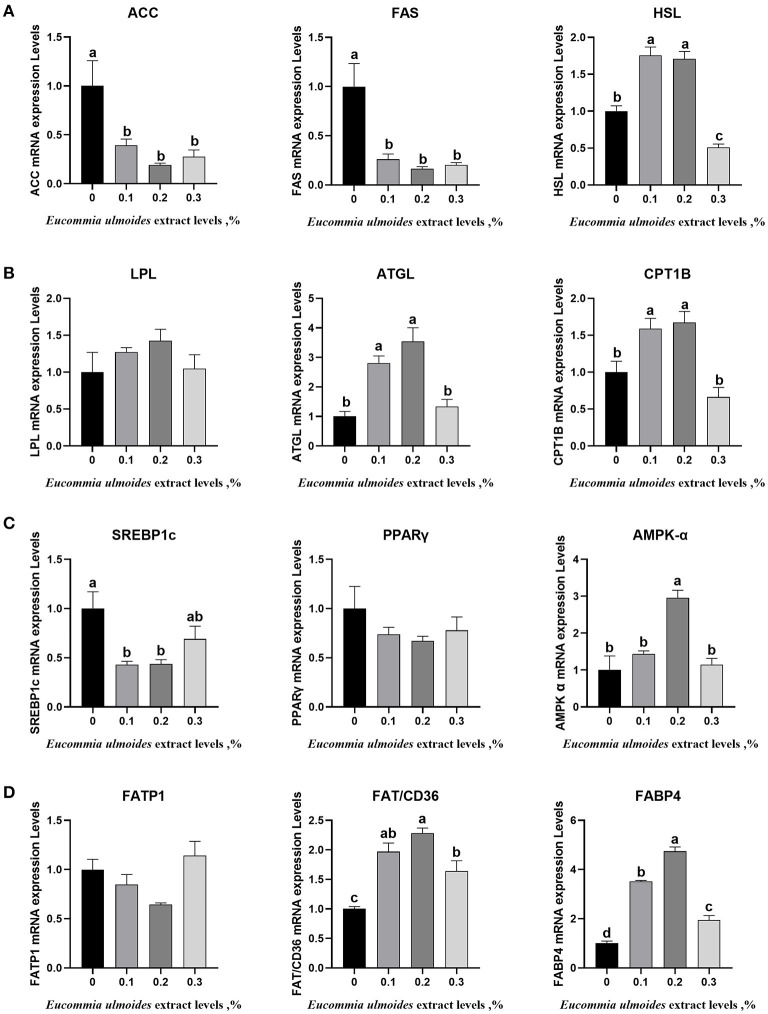
**(A)** The relative mRNA expression levels of the key genes related to lipogenesis including acetyl-CoA carboxylase α (ACC), fatty acid synthase (FAS), and sterol regulatory element-binding protein-1c (SREBP1c), *n* = 8. **(B)** The relative mRNA expression levels of the key genes related to lipolysis including HSL, LPL, and ATGL, *n* = 8. **(C)** The relative mRNA expression levels of the key genes related to fatty acid oxidation including CPT1B, translocase peroxisome proliferator-activated receptor γ (PPARγ), and adenine monophosphate-activated protein kinase α (AMPK-α), *n* = 8. **(D)** The relative mRNA expression levels of the key genes related to fatty acid transport including FATP1, FAT/CD36, and FABP4, *n* = 8. Data are expressed as means ± SEM (*n* = 8). ^a−d^Values with different letters are significantly different among dietary ELE treatments (*p* < 0.05).

Figure 2 shows that dietary supplementation with 0.1% and 0.2% ELE could downregulate (*p* < 0.05) the mRNA expression levels of adipogenic genes such as ACC, FAS and SREBPS1c (Figure 2A) and upregulate (*p* < 0.05) the mRNA expression levels of lipid-lowering genes, such as HSL, ATGL and SREBP1c, but there was no dramatic variation (*p* > 0.05) in LPL in this study (Figures 2A–C). In the 0.2% ELE group, the mRNA expression levels of CPT1 and AMPK-α increased significantly (*p* < 0.05), but that of PPARγ did not change dramatically (*p* > 0.05) (Figures 2B,C). In addition, compared with the control group, supplementing with ELE markedly increased (*p* < 0.05) the mRNA expression levels of FAT/CD36 and FABP4, and 0.2% ELE decreased the mRNA expression level of FATP1 (Figure 2D).

### Western Blotting Analysis

**Figure 3:**
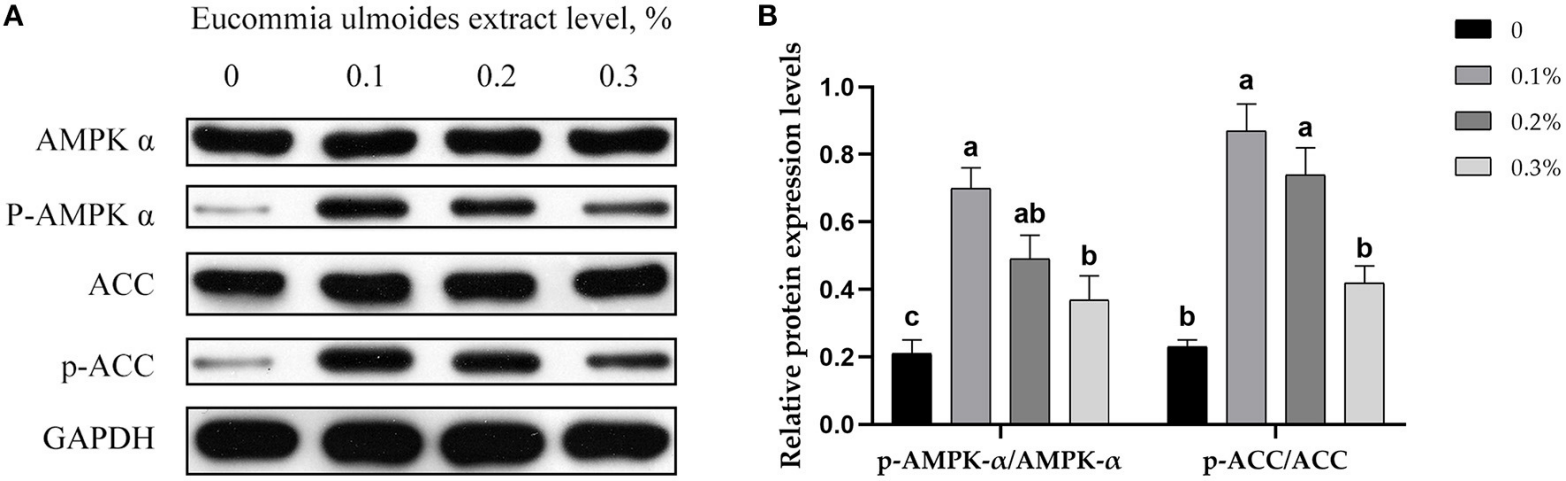
**(A)** Representative immunoblots of protein levels and phosphorylation degrees of AMPK-α, p-AMPK-α, ACC, and p-ACC, in backfat tissue of growing–finishing pigs. **(B)** Relative protein expression levels of p-AMPK-α/AMPK-α and p-ACC/ACC. Data are expressed as means ± SEM. ^a−c^Values with different letters are significantly different among dietary ELE treatments (*p* ≤ 0.05) (*n* = 4).

Relative protein expression levels for AMPK-α, p-AMPK-α, ACC, and p-ACC were determined by using Western blotting. The results showed that all three different levels of ELE upregulated (*p* < 0.05) the relative protein expression levels of p-AMPK-α and p-ACC (Figures 3A,B).

## Discussion

Previous studies have shown that ELE is rich in amino acids, minerals, and other nutrients. The essential amino acid content in ELE is high, of which leucine is the highest, followed by valine (5). In addition, iridoids, phenols, and flavonoids are abundant in ELE, which reduces blood lipids (17, 18) and improves diabetes (19) and antioxidation (7). In recent years, *Eucommia ulmoides* is considered to be a very useful feed additive in healthy livestock and poultry breeding.

This study compared the effects of different supplementary levels of ELE in the diet on growth performance, carcass traits, and lipid metabolism in pigs. Growth performance directly affects the meat growth performance of growing–finishing pigs, thus affecting the economic return. These results showed that supplementing pig diets with different levels of ELE had no significant effect on ADG, ADFI, or F/G in piglets, which was consistent with a previous study (9). This might have been due to the strong aromatic compounds in *E. ulmoides* leaf, which might have affected the palatability of the feed. However, our results differ from some previous studies (10, 11), which might have been due to different processing technologies and amounts of ELE supplements used, while ELE supplements had no negative effects on growth performance.

Human consumption of meat products containing a large amount of fat may pose a threat to health; long-term consumption may induce cardiovascular diseases and obesity (20). Compared with the control group, the average backfat thickness of the three treatment groups decreased by 2.7, 4.6, and 7.4%, respectively. Meanwhile, fat mass percentage in the three treatment groups decreased by 5.1, 3.2, and 1.2%, respectively. Unfortunately, none of them reached a significant level. However, the histomorphological analysis of backfat tissue showed that ELE significantly reduced the average cross-sectional area of adipocyte; the more mature a fat cell is, the larger it is (21), indicating that ELE effectively inhibited the growth and maturation of adipose cells. In conclusion, ELE had a potential inhibitory effect on fat accumulation in back fat tissue; this might be related to chlorogenic acid, the most important active ingredient in ELE. Dietary supplementation with 0.5% and 1% chlorogenic acid was previously reported to reduce the accumulation of visceral fat and lipid content in rats (22). Moreover, dietary supplementation with 0.2% ELE significantly increased carcass weight, slaughter rate, carcass straight length, and loin–eye area, which was consistent with our expectations, indicating that low-dose ELE could improve the carcass traits of growing–finishing pigs.

The changes in serum biochemical indexes can affect the metabolism and nutrient deposition of animals and are affected by the growth stage, endocrine status, and dietary nutrient level (23). We examined the indexes related to lipid and nitrogen metabolism in serum of growing–finishing pigs. Our results showed that adding ELE to the diet increased the serum ALB content and decreased the TP content in growing–finishing pigs, indicating that ELE was beneficial to the overall health of pigs. HDL, a “vascular scavenger,” has an anti-atherosclerosis function (24) and can prevent coronary heart disease (25). VLDL is known as an atherogenic factor. It is reported that *Eucommia ulmoides* reduce the levels of triglycerides and total cholesterol in the plasma of finishing pigs (9). In this study, ELE markedly improved HDL content and decreased the levels of VLDL and TC, which indicated that ELE effectively improved lipid metabolism and cardiovascular health in growing–finishing pigs and that the chlorogenic acid and geniposidic acid contained in ELE could play a vital role in antiobesity. Studies have shown that both chlorogenic acid and geniposidic acid from ELE reduce serum TG and TC in obese mice (17, 18).

Adiponectin is secreted by adipocytes and has insulin-sensitizing, anti-atherosclerotic, and anti-inflammatory effects (26). Previous studies have shown that adiponectin promotes the oxidation of fatty acids in muscle and adipose tissue (27). Adiponectin can increase HDL levels and decrease TG levels (28). IGF-1 is a hormone that is closely related to metabolic syndrome and is mainly secreted by the liver cells. This is related to carbohydrate and lipid metabolism (29). Recombinant IGF-1 enhances the lipolysis of adipose tissue, increases the rate of lipid oxidation (30), and promotes the use of free fatty acids in muscle (29). In this study, supplementation with ELE boosted the concentrations of adiponectin and IGF-1 in circulation, which indicates that it plays a lipid-lowering role by regulating hormone levels in growing–finishing pigs.

To explore whether ELE had a similar effect on enzymes related to lipid metabolism, we measured the activities of enzymes related to lipid metabolism in the serum of fattening pigs. AAC is a well-known rate-limiting enzyme (31–33). HSL and ATGL are two important lipases in the animal body. HSL can hydrolyze TG, diglyceride, monoglyceride, cholesterol ester, retinol ester, and other lipids and produce glycerol and free fatty acids (34, 35). ATGL is highly expressed in adipose tissue and is highly specific for TG (36). Moreover, LPL is a rate-limiting enzyme for the degradation of blood triglycerides to glycerol and free fatty acids (37). In addition, ACAT is the only enzyme in the body that can catalyze cholesterol to produce cholesterol esters. Excessive cholesterol esters may lead to atherosclerosis (38). In the present study, although ELE did not dramatically change ACAT activity, dietary supplementation with 0.1% ELE increased HSL, LPL, and ATGL activities while decreasing ACC activity, enlightening the effect of ELE on reducing serum TG which could be achieved by regulating the activities of these enzymes. In addition, we also analyzed the histomorphology of backfat, and the results showed that the addition of ELE markedly reduced the mean cross-sectional area of adipocyte and increased the number of backfat adipocytes. It is proved that ELE can inhibit fat deposition.

We further examined the mRNA expression levels of enzymes and cytokines related to lipogenesis (ACC and FAS), lipolysis (HSL, LPL, and ATGL), fatty acid oxidation (CPT1B, AMPK α), fatty acid transportation (FATP1, FAT/CD36, and FABP4), and lipid deposition (SREBP-1c, PPARγ) to determine the molecular mechanism by which ELE regulates lipid metabolism in growing–finishing pigs. In this study, all three levels of ELE downregulated the mRNA expression of ACC and FAS, and 0.2% ELE significantly downregulated the expressions of SREBP1c and FATP1. The level of PPARγ mRNA showed a downward trend but did not reach a memorable level. These results showed that 0.1 and 0.2% ELE could effectively reduce the mRNA expression levels of adipogenesis genes. In addition, supplementing with 0.1 and 0.2% ELE upregulated the mRNA level expressions of HSL, ATGL, CPT1B, AMPK, FAT/CD36, and FABP4. Compared with the control group, the 0.3% ELE supplement also significantly upregulated the expressions of FAT/CD36 and FABP4 mRNA. Additionally, the 0.3% ELE supplement downregulated the mRNA expression level of HSL, suggesting that the lipid-lowering effect of ELE may decrease when the dosage exceeds 0.2%. The mRNA expression levels of HSL and ATGL were consistent with the observations that ELE increased HSL and ATGL enzyme activities in growing–finishing pigs. These results also reveal that ELE can exert a lipid-lowering effect by downregulating the mRNA expression levels of lipid-producing genes and upregulating the mRNA expression levels of lipid-lowering genes.

AMPK and ACC are not only the key links in their metabolic regulation but also closely related to each other, which can form upstream and downstream signal pathways in cells. The AMPK-ACC signaling pathway formed by AMPK and its downstream target ACC has important physiological significance in the process of fat synthesis and oxidation (39). When activated by adiponectin, AMPK phosphorylation inactivates ACC phosphorylation, which catalyzes the formation of malonyl-CoA. Malonyl-CoA is the substrate for fatty acid biosynthesis, which inhibits fatty acid oxidation (40). Therefore, we speculate that ELE has a lipid-lowering effect through the AMPK-ACC pathway. We measured the protein expression level of AMPK-α and ACC and the expression level of phosphorylated proteins. As expected, all the three levels of ELE remarkably improved the protein levels of p-AMPK-α and p-ACC and showed a downward trend with the increase in dosage.

## Conclusions

The addition of ELE <0.3% in growing–finishing pigs could partially improve the carcass traits of growing–finishing pigs and had no adverse effect on growth performance. The 0.1% ELE supplement improved carcass traits, and the 0.1 and 0.2% ELE supplement can reduce the level of TG in serum and increase the level of hormones and enzyme activity that promote fat catabolism. The mRNA and protein expression levels of the key genes related to lipid metabolism showed that the lipid-lowering mechanism of ELE may be through the activation of the AMPK-ACC pathway to inhibit fat deposition in backfat tissue, and the lipid-lowering effect of the 0.1 and 0.2% ELE supplement was the best. However, when the supplemental level was 0.3%, there was no significant effect on carcass traits and lipid metabolism of growing–finishing pigs, and some indexes even had negative effects. In conclusion, the supplemental range of 0.1 to 0.2% ELE is the optimal addition. ELE contains a variety of bioactive components; which component plays a leading role needs our further study using the cell culture model.

## Data Availability Statement

The datasets presented in this study can be found in online repositories. The names of the repository/repositories and accession number(s) can be found in the article/supplementary material.

## Ethics Statement

The animal study was reviewed and approved by Committee on Animal Care of the Institute of Subtropical Agriculture, Chinese Academy of Sciences.

## Author Contributions

YuhY, FL, QG, and YulY designed the experiments. YuhY and YunY conducted the experiments. WW, QG, LZ, YunY, MH, and SG helped with animal experiments. YuhY analyzed the data and wrote the original draft. QG and FL revised the manuscript. All authors have read and approved the final manuscript.

## Funding

This work was funded by the National Nature Science Foundation of China (31972582), the Science and Technology Innovation Program of Hunan Province (2021RC4039), Funds for Distinguished Young Youths of Hunan Province (2020JJ2030), Key R&D Program of Hunan Province (2022NK2026), the Science and Technology Projects of Changsha City (kq1801059), the Youth Innovation Promotion Association CAS (Y202079), the Earmarked Fund for China Agriculture Research System (CARS-35), and the Open Fund of Key Laboratory of Agro-Ecological Processes in Subtropical Region, Chinese Academy of Sciences (No. ISA2021202).

## Conflict of Interest

The authors declare that the research was conducted in the absence of any commercial or financial relationships that could be construed as a potential conflict of interest.

## Publisher's Note

All claims expressed in this article are solely those of the authors and do not necessarily represent those of their affiliated organizations, or those of the publisher, the editors and the reviewers. Any product that may be evaluated in this article, or claim that may be made by its manufacturer, is not guaranteed or endorsed by the publisher.
